# Validation of the World Health Organization/ International Society of Hypertension (WHO/ISH) cardiovascular risk predictions in Sri Lankans based on findings from a prospective cohort study

**DOI:** 10.1371/journal.pone.0252267

**Published:** 2021-06-07

**Authors:** U. B. Thulani, K. C. D. Mettananda, D. T. D. Warnakulasuriya, T. S. G. Peiris, K. T. A. A. Kasturiratne, U. K. Ranawaka, S. Chakrewarthy, A. S. Dassanayake, S. A. F. Kurukulasooriya, M. A. Niriella, S. T. de Silva, A. P. Pathmeswaran, N. Kato, H. J. de Silva, A. R. Wickremasinghe

**Affiliations:** 1 Department of Public Health, Faculty of Medicine, University of Kelaniya, Ragama, Sri Lanka; 2 Department of Pharmacology, Faculty of Medicine, University of Kelaniya, Ragama, Sri Lanka; 3 Department of Physiology, Faculty of Medicine, University of Kelaniya, Ragama, Sri Lanka; 4 Department of Mathematics, Faculty of Engineering, University of Moratuwa, Moratuwa, Sri Lanka; 5 Department of Medicine, Faculty of Medicine, University of Kelaniya, Ragama, Sri Lanka; 6 Department of Biochemistry, Faculty of Medicine, University of Kelaniya, Ragama, Sri Lanka; 7 National Center for Global Health and Medicine, Toyama, Shinjuku-ku, Tokyo, Japan; Universidad Miguel Hernandez de Elche, SPAIN

## Abstract

**Introduction and objectives:**

There are no cardiovascular (CV) risk prediction models for Sri Lankans. Different risk prediction models not validated for Sri Lankans are being used to predict CV risk of Sri Lankans. We validated the WHO/ISH (SEAR-B) risk prediction charts prospectively in a population-based cohort of Sri Lankans.

**Method:**

We selected 40–64 year-old participants from the Ragama Medical Officer of Health (MOH) area in 2007 by stratified random sampling and followed them up for 10 years. Ten-year risk predictions of a fatal/non-fatal cardiovascular event (CVE) in 2007 were calculated using WHO/ISH (SEAR-B) charts with and without cholesterol. The CVEs that occurred from 2007–2017 were ascertained. Risk predictions in 2007 were validated against observed CVEs in 2017.

**Results:**

Of 2517 participants, the mean age was 53.7 year (SD: 6.7) and 1132 (45%) were males. Using WHO/ISH chart with cholesterol, the percentages of subjects with a 10-year CV risk <10%, 10–19%, 20%-29%, 30–39%, ≥40% were 80.7%, 9.9%, 3.8%, 2.5% and 3.1%, respectively. 142 non-fatal and 73 fatal CVEs were observed during follow-up. Among the cohort, 9.4% were predicted of having a CV risk ≥20% and 8.6% CVEs were observed in the risk category. CVEs were within the predictions of WHO/ISH charts with and without cholesterol in both high (≥20%) and low(<20%) risk males, but only in low(<20%) risk females. The predictions of WHO/ISH charts, with-and without-cholesterol were in agreement in 81% of subjects (ĸ = 0.429; p<0.001).

**Conclusions:**

WHO/ISH (SEAR B) risk prediction charts with-and without-cholesterol may be used in Sri Lanka. Risk charts are more predictive in males than in females and for lower-risk categories. The predictions when stratifying into 2 categories, low risk (<20%) and high risk (≥20%), are more appropriate in clinical practice.

## Introduction

Asians are reported to have a different cardiovascular disease (CVD) profile than those in western countries. Asians have more strokes than coronary heart diseases (CHD), tend to have CVDs at younger ages, and develop CVDs despite having low serum cholesterol levels and body mass indexes (BMIs) compared to Caucasians [1–3]. South Asians including Sri Lankans have a distinct genetic make-up, a higher prevalence of hypertension, diabetes mellitus, central obesity, insulin resistance, and metabolic syndrome than whites in the UK and America [2, 4–9]. South Asians also have a higher salt intake and are undergoing huge lifestyle and socioeconomic transformations, all leading to a high cardiovascular (CV) risk [10–13]. Furthermore, it is reported that their absolute CV risk is higher than the predictions of standard European scores [14].

Cardiovascular risk prediction is important to make treatment decisions especially in primary prevention of CVDs based on a total risk approach. Even though many cardiovascular risk prediction models exist [15], only very few are targeted for Asians [16–18]; none is derived from South Asians including Sri Lankans. Therefore, different risk prediction models such as the World Health Organization/ International Society of Hypertension (WHO/ISH) risk prediction charts [19] and Framingham risk scores (FRS) [20] are being used in Sri Lanka to identify individuals at high risk of CVD.

Literature on the applicability of well-known risk estimates in South Asians is not consistent [21, 22]. An Indian study reported that the Framingham general CVD risk calculator is the best for Indians and the WHO/ISH and Atherosclerotic Cardiovascular Disease risk calculators (ASCVD) performed the worst of many other tests [23]; some reports indicate that even the Framingham based tools underestimate the cardiovascular risk of Indians [24]. The pooled cohort equation is also reported to overestimate the CV risk of Asians [25]. Another study comparing risk equations using “limited information” derived from the Framingham study, when re-calibrated with contemporary data and of Asian cohorts, found that both equations were predictive of future cardiovascular risks with similar accuracy in Asian populations [26].

The WHO/ISH risk prediction charts that have been developed using large samples for different regions of the world which apply to specific countries are widely used. The WHO/ISH risk prediction charts indicate 10-year risk of a fatal or non-fatal major cardiovascular event (myocardial infarction or stroke), according to age, sex, blood pressure, smoking status, total blood cholesterol and the presence or absence of diabetes mellitus for 14 WHO epidemiological sub-regions. There are two sets of charts; one set (14 charts) can be used in settings where blood cholesterol can be measured and the other (14 charts) is for settings in which blood cholesterol cannot be measured [19]. The chart applicable to Sri Lanka is the “Southeast Asia Region B (SEAR B)” chart.

We aimed

1. to compare the risk predictions of WHO/ISH(SEAR B) charts with- and without-cholesterol in a population-based cohort of Sri Lankans, and
2. to compare the 10-year risk predictions of WHO/ISH (SEAR B) charts with observed CVEs in a population-based cohort of Sri Lankans stratified by males and females.

## Methods

### Study design

This longitudinal study comprised baseline data extraction from a larger community-based study on non-communicable diseases, the Ragama Health Study (RHS) [27, 28] and primary data collection to ascertain CVEs over 10 years.

### Study setting

The study was carried out in the Ragama Medical Officer of Health (MOH) area in Sri Lanka. Sri Lanka is divided into 331 MOH areas for purposes of provision of preventive health services. in the country. The RHS was initiated in 2007 in the Ragama MOH area as a collaboration between the International Medical Centre of Japan (IMCJ) and the Faculty of Medicine, University of Kelaniya, Sri Lanka to obtain information on the prevalence of NCDs and risk factors that may be modified to formulate effective and efficient NCD intervention strategies. The Ragama MOH area is one of 16 MOH areas of the Gampaha district of Sri Lanka and is located 18 km north of the business capital of Sri Lanka, Colombo. In 2007, the Ragama MOH area had a multi-ethnic population of 75 591 people with 15 137 housing units extending in an area of 25 km^2^ in an urban to a semi-urban setting. The MOH provides preventive care and public health services to a population in a designated area. These services include maternal and child health, nutrition, environmental and occupational health, control of communicable and non-communicable diseases, food safety, etc. Curative care is provided by a network of public and private health care providers throughout the country; services of the public sector are provided free of charge.

The baseline study population in the RHS comprised 35–64-year-old adults resident in the Ragama MOH area. Using the voters’ list of each Grama Niladhari (GN) division (the smallest administrative unit in Sri Lanka), all 35–64-year-old adults were enumerated and were stratified into three age groups (35–44, 45–54 and 55–64 years). As the aim of the original study was to select a sample of 3 000 persons most likely to have non-communicable diseases or their modifiable risk factors for follow up, it was decided to sample 200 adults each from 16 GN divisions, randomly selected from the 22 GN divisions in the MOH area. Random samples of 200 adults from each of the selected 16 GN divisions, with a ratio of 1:2:2 in the 35–44, 45–54, and 55–64 year age groups were identified from voters’ lists maintained by GN officers using computer-generated random numbers. The ratio of 1:2:2 for the three age groups was taken to maximize the probability of recruiting persons with non-communicable diseases or their risk factors.

The WHO/ISH cardiovascular risk prediction charts are applicable for persons 40–80 years of age. The10-year risk predictions of a fatal or non-fatal cardiovascular event at baseline in 2007 were calculated using WHO/ISH (SEAR B) risk prediction charts with and without cholesterol [19]. The risk models included sex, age, systolic blood pressure, current smoking status, and diabetes mellitus in both models, and in addition, the total cholesterol level in the risk prediction chart with cholesterol. The charts use the following categorizations for 10-year risk predictions: gender male or female; age 40–49, 50–59, 60–69 and 70–79 years; smoking status as smoker or non-smoker; systolic blood pressure 120–139, 140–159, 160–179 and ≥180 mm Hg; total cholesterol 4–4.9, 5–5.9, 6–6.9, 7–7.9 and ≥8 mmol/L; separate charts are available for persons with and without diabetes. The age of the person was cross-checked with the date of birth given in the national identity card. The operational definitions of the risk factors are given in Table 1.

**Table 1:** Definitions of risk factors used in 10-year cardiovascular risk predictions (2007).

Risk factor	Definition
Smokers	All current smokers and those who quit smoking within a year of assessment in 2007.
Diabetics^a^	A person with self-reported diabetes mellitus cross-checked with medical records or taking insulin or oral hypoglycaemic drugs, or with a fasting plasma glucose concentration above 126 mg/dl (>7 mmol/l) according to World Health Organization criteria [19, 29].
Hypertension	A person with self-reported hypertension cross-checked with medical records, physician-diagnosed hypertension, or systolic blood pressure (SBP) ≥ 140 mmHg or diastolic blood pressure (DBP) ≥ 90 mmHg on two different occasions or taking antihypertensive medications, according to Joint National Committee (JNC) VII criteria [30]
Hyperlipidaemia	A person with physician-diagnosed hyperlipidaemia in medical records, a history of taking anti-hyperlipidemic drugs or with a fasting plasma total cholesterol concentration above 200 mg/dl (>5.17 mmol/l) based on National Cholesterol Education Program III criteria [31].
Systolic blood pressure	Mean systolic blood pressure at baseline
Total cholesterol	total cholesterol levels at baseline

^a^ used only for epidemiological purposes

### Study population

Of the subjects enrolled in the main RHS study, the participants above 40 years of age (in 2007) were included in this study as the WHO/ISH risk prediction charts apply only to persons 40–80 years of age. We excluded persons who could not be traced in 2017 or whose current status (dead or alive) could not be ascertained; if a person had died, if the cause of death could not be verified, that person was also excluded.

### Baseline data collection and abstraction

At baseline, all selected individuals were visited at their homes and invited to participate in the study. All were explained about the purpose of the study, the procedures, potential hazards, and benefits of the study, and informed written consent was obtained. All consenting adults were requested to present for an interview, anthropometric measurements, and collection of blood samples following a 12-hour fast with any available health records. Subjects were interviewed by trained medical graduates and data were collected using a questionnaire to obtain information on socio-demographic characteristics and past medical and drug history. All past medical records of the subjects were reviewed, and relevant information recorded. Anthropometric measurements were recorded using standard procedures and BMI was calculated. Blood pressure (BP) was measured using an Omron 705CP automated blood pressure monitor in the left arm in the seated position. The mean of two BP measurements, taken 5 minutes apart was defined as BP. A10-mL sample of venous blood was obtained from each subject to measure fasting blood sugar (FBS) and serum lipids (total cholesterol (TC), low-density lipoprotein (LDL) cholesterol, high-density lipoprotein (HDL) cholesterol and triglycerides (TG). Plasma glucose was measured according to the hexokinase method and total cholesterol by the method described by Stadtman, and LDL cholesterol by the homogenous assay method using Automated LDL Cholesterol (ALDL)-Flex reagent cartridges(Dimension Clinical Chemistry System, Dade Behring, USA) [32].

Baseline data on age, sex, systolic blood pressure, smoking status, total blood cholesterol and presence or absence of diabetes mellitus used for risk prediction were abstracted. Subjects were categorised into 5 risk-levels according to WHO/ISH risk charts using these baseline results; <10%, 10–19%, 20%-29%, 30–39%, and ≥40%. Subjects were further categorized into 2 broad risk groups; low risk (<20%) and high risk (≥ 20%) for practical use in primary care.

### Follow up and primary data collection

The same cohort was followed up in 2010, 2014, and 2017 as part of the larger study. In 2010 and 2014, participants were contacted via telephone and home visits and requested to present themselves for an interview and follow up examination during which anthropometric measurements were recorded and blood assays were done. In 2017, house visits were done to ascertain their current status. When the family of the participant was not resident in the given address, attempts were made to contact them through telephone if a telephone number was available. If telephone contact was not possible, then the neighbouring houses were visited and information, if any, of the family was obtained and attempts were made to contact the participants.

All cardiovascular deaths, non-fatal strokes, and non-fatal myocardial infarctions including elective percutaneous coronary interventions and coronary artery bypass grafts done on patients with symptomatic unstable angina that occurred from 2007 to 2017 were recorded as cardiovascular events during these follow-up visits by interviewing patients and their families and perusing clinical notes/death certificates.

### Data management and analysis

Baseline data were extracted from Excel files. Data collected in 2017 were entered into excel files and manually checked. All data files were exported into SPSS version 22 and merged using the unique identification number.

All statistical analyses were done using SPSS version 22. Categorical data are reported as percentages. Continuous variables are reported as means with standard deviation (SD) or 95% confidence intervals. The significance level was set at p <0.05. Associations between subgroups and risk levels were tested using the chi-square test.

As the WHO/ISH risk prediction charts give the risk of a fatal or non-fatal cardiovascular event over10 years, we compared the observed events with the predicted risk for the events. Only the first-CVD or death during the follow-up period for a specific patient was counted as a new CV event for observed events; recurrent events during the follow-up period were not counted as new CV events. The cohort was initially categorized in to predicted risk categories; 1) to five risk categories (<10%, 10–19%, 20%-29%, 30–39%, ≥40%), and 2) to two risk categories (< 20% and ≥20%) based on their predicted risk calculated using WHO/ISH risk charts with- and without-cholesterol. For example, among those with a predicted risk of <10%, we first confirmed whether the observed risk was less than 10%. If the observed risk was more than 10%, we then checked if the lower limit of the 95% confidence interval of the observed risk included 10%. A similar analysis was done for the other predicted risk groups as well. We considered that the predicted and observed risks were similar if the 95% confidence interval of the observed risk included the upper or lower limit of the predicted risk category. The risk predictions of the two charts with- and without-cholesterol were compared using the kappa statistic.

### Ethics approval

Ethics approval was obtained from the Ethics Review Committee of the Faculty of Medicine, University of Kelaniya, Sri Lanka (P38/09/2006). This approval covered additional follow up investigations and data collection of participants included in the original cohort. Written informed consent of participants was obtained for the follow-up interviews.

## Results

Of the 2923 participants enrolled at the beginning of the RHS study, 2 685 were followed-up after 10 years while the remaining 238 could not be traced. Of the 2685 who were followed-up at after 10 years, 2517 had complete data for risk prediction and were included in the study. The group lost to follow-up was not significantly different from the group followed in terms of gender, age, current smoking status or FBS, SBP, or total cholesterol level at baseline (S1 Table).

The demographic characteristics and past medical history of the study cohort are given in Table 2. There were 1132 (45.0%) males and the mean age (±SD) of the cohort at baseline was 53.7 ± 6.7 years. The majority (96.2%) was Sinhalese and 54.9% were educated up to 10 years or above. Baseline risk factor distribution among participants in 2007 is shown in Table 2.

**Table 2:** Baseline characteristics of participants.

	Male	Female	Total
	n = 1132	n = 1385	n = 2517
**Ethnicity n (%)**^**a**^			
Sinhalese	1088 (96.3)	1318 (96.1)	2406 (96.2)
Tamils	11 (1.0)	24 (1.8)	35 (1.4)
Muslims	2 (0.2)	2 (0.1)	4 (0.2)
Burghers	15 (1.3)	17 (1.2)	32 (1.3)
Other	14 (1.2)	11 (0.8)	25 (0.9)
**Level of education n (%)**^**b**^^,^^**c**^			
< 5 years of schooling	55 (4.9%)	126 (9.3%)	181 (7.3)
5–10 years of schooling	403 (35.9%)	534 (39.3%)	937 (37.8)
11 years of schooling	442 (39.4%)	447 (32.9%)	889 (35.8)
13 years of schooling	165 (14.7%)	215 (15.8%)	380 (15.3)
Diploma/ graduate/ postgraduate	57 (5.1%)	37 (2.7%)	94 (3.8)
**Age groups (years),** n(%)			
40–49.9	341 (30.1)	422 (30.5)	763 (30.3)
50–59.9	518 (45.8)	645 (46.6)	1163 (46.2)
≥ 60.0	273 (24.1)	318 (23.0)	591 (23.5)
**Smoking,** n(%)	395 (34.9%)	0 (0.0)	395 (15.7%)
**Diabetes mellitus,** n(%)	457 (40.4%)	552 (39.9%)	1009 (40.1%)
**Hyperlipidemia,** n(%)	130 (11.5)	226 (16.3)	356 (14.1)
**SBP (mm Hg),** n(%)		
<139.9	732 (64.7%)	817 (59.0%)	1549 (61.5%)
140–159.9	263 (23.2%)	362 (26.1%)	625 (24.8%)
160–179.9	90 (8.0%)	133 (9.6%)	223 (8.9%)
≥180.0	47 (4.2%)	73 (5.3%)	120 (4.8%)
**Total cholesterol (mmol/l),** n(%)			
<4.0	463 (40.9%)	393 (28.4%)	856 (34.0%)
5–5.9	399 (35.2%)	497 (35.9%)	896 (35.6%)
6–6.9	198 (17.5%)	335 (24.2%)	533 (21.2%)
7–7.9	67 (5.9%)	123 (8.9%)	190 (7.5%)
≥8.0	5 (0.4%)	37 (2.7%)	42 (1.7%)
**BMI**≥**23Kg/m**^**2**^, n(%)	916 (66.1)	578 (51.1)	1494 (59.4)
**BMI**≥**30Kg/m**^**2**^, n(%)	47 (4.2)	160 (11.6)	207 (8.2)

^a^ missing data n = 15

^**b**^ years of schooling

^c^ missing data n = 36

Comparison of the risk predictions of the two models of WHO/ISH risk charts is shown in Table 3. The risk predictions of the two charts agreed in 2033 out of 2517 (80.3%) (ĸ = 0.429; p<0.001) (highlighted in Table 3) participants by being classified in the same risk categories by both risk charts. However, the agreement was poor in higher-risk categories.

**Table 3:** Comparison of WHO/ISH 10-year CV risk predictions made using charts with and without cholesterol^a^.

10-year CV risk prediction (WHO/ISH charts without-cholesterol)	10-year CV risk prediction (WHO/ISH charts with cholesterol) n (%)					Total
	< 10%	10–19%	20–29%	30–39%	≥ 40%	
**< 10%**	1866 (74.13%)	107 (4.25%)	36 (1.43%)	5 (0.19%)	1 (0.04%)	2015 (80.06%)
**10–19%**	165 (6.56%)	140 (5.56%)	38 (1.51%)	27 (1.07%)	26 (1.03%)	396 (15.73%)
**20–29%**	1 (0.04%)	3 (0.12%)	20 (0.79%)	26 (1.03%)	43 (1.71%)	93 (3.69%)
**30–39%**	0 (0.0%)	0 (0.0%)	0 (0.0%)	0 (0.0%)	0 (0.0%)	1 (0.04%)
**≥ 40%**	0 (0.0%)	0 (0.0%)	1 (0.04%)	7 (0.28%)	7 (0.28%)	12 (0.48%)
Total	2032 (80.73%)	250 (9.93%)	96 (3.82%)	62 (2.46%)	77 (3.06%)	2517 (100)

^a^ Kappa statistic (ĸ) = 0.429; p<0.001

A total of 168 deaths were reported over the 10-year follow-up, of which, 73 were due to cardiovascular causes. A total of 142 non-fatal CV events also occurred during the same period. Of them, 115 were cardiac events (including myocardial infarctions, coronary artery bypass grafts (CABG), and percutaneous coronary interventions (PCI) following unstable angina) and 27 were strokes. The total cardiovascular events, both fatal and non-fatal, reported were 215. The majority of the events were in males; 130/215 (60.5%).

The observed CV events rate over the 10 years was 8.5% (215/2517). The majority of the cohort was at low risk (<20%) of CV events; 90.6% and 95.2%when using WHO/ISH charts with- and without-cholesterol, respectively. Validation of the risk predictions of the two versions of WHO/ISH (SEAR B) charts against observed CV events is shown in Tables 4 and 5 separately. The observed risks matching the predicted risks are highlighted. Risk predictions of both WHO/ISH (SEAR B) charts with- and without-cholesterol were in agreement with percentages of observed events among both low risk (<20%) and high risk (≥20%) participants (highlighted in Tables 4 and 5) except in females when using the chart with-cholesterol (Table 4). The high-risk prediction for females (≥20%) using the WHO/ISH (SEAR B) chart with-cholesterol over-estimated their observed risk which was 15.3% (12.5–18.2%) (Table 4).

**Table 4:** Validation of WHO/ISH (SEAR B) risk prediction charts with cholesterol over 10 years.

10-year CV risk prediction (WHO/ISH charts with-cholesterol)	Male		Female		Total	
	n	Observed CV events N (%) [95% confidence interval]	n	Observed CV Events N (%) [95% confidence interval]	n	Observed CV Events N (%) [95% confidence interval]
**<10%**	987	94 (9.5) [8.6–10.5%]	1045	48 (4.6) [3.9–5.2%]	2032	142 (7.0) [6.4–7.6%]
**10–19%**	73	19 (26.0) [20.9–31.2%]	177	12 (6.8) [4.9–8.7%]	250	31 (12.4) [10.3–14.5%]
**20–29%**	41	6 (14.6) [9.1–20.2%]	55	6 (10.9) [6.7–15.1]	96	12 (12.5) [9.1–15.9%]
**30–39%**	14	4 (28.6) [16.5–40.6%]	48	8 (16.7) [11.3–22.0%]	62	12 (19.4) [14.3–24.4%]
**≥40%**	17	7 (41.2) [29.2–53.1%]	60	11 (18.3) [13.3–23.3%]	77	18 (23.4) [18.6–28.2%]
**<20%**	1060	113 (10.6) [9.7–11.6%]	1222	60 (4.9) [4.3–5.5%]	2282	173 (7.6) [7.0–8.1%]
**≥20%**	72	17 (23.6) [18.6–28.6%]	163	25 (15.3) [12.5–18.2%]	235	42 (17.9) [15.4–20.4%]
**Total**	1132	130 (11.5) [10.5–12.4%]	1385	85 (6.1) [5.5–6.8%]	2517	215 (8.5) [8.0–9.1%]

**Table 5:** Validation of WHO/ISH (SEAR B) risk prediction charts without cholesterol over 10 years.

10-year CV Risk prediction (WHO/ISH charts without cholesterol)	Male		Female		Total	
	n	Observed CV events N (%) [95% confidence interval]	n	Observed CV events N (%) [95% confidence interval]	n	Observed CV events N (%) [95% confidence interval]
**<10%**	931	89 (9.6) [8.6–10.5%]	1084	49 (4.5) [3.9–5.2%]	2015	138 (6.8) [6.3–7.4%]
**10–19%**	157	28 (17.8) [14.8–20.9%]	239	26 (10.9) [8.9–12.9%]	396	54 (13.6) [11.9–15.4%]
**20–29%**	31	10 (32.3) [23.9–40.7%]	62	10 (16.1) [11.5–20.8%]	93	20 (21.5) [17.2–25.8%]
**30–39%**	1	0 (0.0)	0	0 (0.0)	1	0 (0.0)
**≥40%**	12	3 (25.0) [12.5–37.5%]	0	0 (0.0)	12	3 (25.0) [12.5–37.5%]
**<20%**	1088	117 (10.8) [9.8–11.7%]	1323	75 (5.7) [5.0–6.3%]	2411	192 (8.0) [7.4–8.5%]
**≥20%**	44	13 (29.5) [22.7–36.4%]	62	10 (16.1) [11.5–20.8%]	106	23 (21.7) [17.7–25.7%]
**Total**	1132	130 (11.5) [10.5–12.4%]	1385	85 (6.1) [5.5–6.8%]	2517	215 (8.5) [8.0–9.1%]

## Discussion

This is the first study to validate WHO/ISH risk prediction charts in a South Asian / Sri Lankan population. We found that the WHO/ISH(SEAR B) risk charts provide good 10-year CV-risk predictions for Sri Lankans and that the predictions using the two charts, with- and without-cholesterol, were in agreement (ĸ = 0.429; p<0.001). The predictions of both charts were good for the risk categories <10% and <20%, overall, and for males and females separately. The chart using cholesterol correctly predicted all risk categories among males except for the 10–19% risk category. The chart without-cholesterol correctly predicted all risk categories overall and for males and females, except for the risk categories of 30–39% and ≥40%. When risk was stratified into low risk (<20%) and high risk (≥20%), all predictions, including overall, males and female, of both charts were correct except the predictions for high-risk females (≥20%) when the charts with cholesterol were used.

We observed that the predictions using the two charts, with- and without-cholesterol, were in moderate agreement (81%). The main objective of developing the chart without-cholesterol was for risk stratification in resource-poor settings where total cholesterol is not freely available. The results of our study are similar to a study done in India [33].

We found that the WHO/ISH(SEAR B) risk charts provide accurate 10-year CV-risk predictions in this Sri Lankan cohort among those categorised as having risks of <10% and <20%. The better accuracy in these risk categories is probably due to the relatively large sample sizes in these categories which would have stabilised the predicted risks. The sample sizes in the other risk categories were small; this is evident by the wide confidence intervals of the estimates.

The chart with cholesterol accurately predicted the CV risk among males in all risk categories except the 10–19% risk category; the accuracy of prediction among females was good only for the <10% and <20% risk categories. In the chart without-cholesterol, the accuracy of the predictions for males and females in different risk categories were similar; the predictions were poor for the risk categories 30–39% and ≥40%. The risk predictions for males have been reported to be better in a multiethnic Asian population (including Malays, Chinese, and Indians) that was stratified using the Framingham General CVD Risk score [34]. It is well known that the risk factors, pathologies, disease patterns, and outcomes of CVDs are different in males and females [35–37]. To address this, the WHO/ISH charts have separate charts for males and females. Despite having separate charts, the risk predictions of females were less accurate than in males probably because of sex-specific risk factors such as menopause, hormonal imbalance due to premature ovarian failure or use of hormones, pregnancy complications, polycystic ovary syndrome, etc. not being considered in the WHO/ISH risk prediction charts [38, 39].

We observed that the WHO/ISH (SEAR B) charts are more accurate and more informative in categorizing patients into “low”(<20%) and “high” (≥) risk groups for clinical practice. Risk prediction is important in detecting high-risk persons to target primary preventive measures under the total cardiovascular risk approach. The WHO/ISH risk prediction charts have been developed using a modelling approach with best available data on risk factor prevalences, population incidence rates and relative risks at different WHO epidemiologic sub-regions studied by the Comparative Risk Assessment Project of WHO [40] and the Asia Pacific Cohort Studies Collaboration [3].

In this study, the high-risk population varied between 4.2–9.3% based on the two WHO/ISH (SEAR B) charts. Our sample was a representative sample of an urban/suburban population where CV risk factors are more likely to be concentrated than in a rural population due to rapid change to a westernized lifestyle. Ranawaka et al. reported the same to be 10.7% in 2007 in the same population with a larger sample size (n = 2985) including persons 35 years and above [28]. Mendis et al. reported a lower prevalence of 1.7% using the same WHO/ISH (SEAR B) charts in a sample of 1000 randomly selected 40–80 year-olds representing the general population of Sri Lanka in 2011 in a multi-country study including Nigeria, Iran, China, Pakistan, Georgia, Nepal, Cuba, and Sri Lanka [41]. However, the Sri Lankan population studied has not been specified.

Around 80% of the global cardiovascular epidemiology is reported from low middle-income countries (LMIC) [42]. Being a LMIC, primary prevention of CVD is a priority for Sri Lankans. Our findings endorse the possibility of using WHO/ISH (SEAR B) chart without-cholesterol for risk stratification in rural Sri Lanka. We propose that for practical purposes, the cut-off value for low and high-risk differentiation of Sri Lankans should be set at <20/≥20% rather than at <30/≥30% which was the suggested by Mendis et al. [41]. It is emphasized that the CV risk may be higher than the predicted risk in patients who are already on antihypertensive therapy or are approaching a higher categorisation criterion, had premature menopause, central obesity, sedentary lifestyle, a family history of premature CVD, high triglyceride or low HDL levels, impaired glucose tolerance, microalbuminuria or socioeconomic deprivation etc. [43]. Furthermore, it is important to consider patients who have already had previous CVDs or with very high individual risk factor levels; persistently raised blood pressure ≥160/100 mm Hg, total cholesterol ≥8 mmol/l, or established diabetes mellitus with renal disease as having high CV risk, irrespective of the WHO/ISH chart risk predictions in deciding on preventive and treatment modalities [19, 44].

Our study has many strengths. This is a randomly selected, population-based prospective study, having individual patient follow-up data for 10 years. The dropout rate was very low. Patients were followed up almost every three years during the 10 years with meticulous care in documenting events based on available evidence ensuring accurate data. Self-reported bias is unlikely as we checked available medical records at baseline and in determining CV outcomes. We are confident that the predictions based on this study are accurate as they are based on a 10-year follow up using stringent measures to ascertain outcomes. Also, the use of actual data to validate risk predictions of the WHO/ISH (SEAR B) charts without using a modelling approach is a strength. However, there are a few limitations of our study. We studied an urban/suburban Sri Lankan population and therefore, the exact risk predictions may not be generalizable to all Sri Lankans.

## Conclusions

WHO/ISH (SEAR B) risk prediction charts with- and without-cholesterol are satisfactory for CV risk stratification of Sri Lankans. The risk predictions of the two WHO/ISH (SEAR B) risk prediction charts, with- and without-cholesterol, appear to agree. The risk predictions of the two WHO/ISH (SEAR B) risk prediction charts, with- and without-cholesterol, can be used depending on the availability of resources. Risk charts are more predictive in males than in females and for lower-risk categories. The predictive value is best when stratifying into 2 categories, low risk (<20%) and high risk (≥20%), which is more appropriate in clinical practice.

## Supporting information

S1 Table “Comparison of baseline characteristics in 2007 of the study population and the group lost to follow-up in 2017”. ^1^ p-value-based on the chi-square, ^2^ p-value-based on the independent sample t-test.
(DOCX)
