# Supplementary material for: Validation of the World Health Organization/ International Society of Hypertension (WHO/ISH) cardiovascular risk predictions in Sri Lankans based on findings from a prospective cohort study
Source: PLoS One. 2021 Jun 7;16(6):e0252267. doi: 10.1371/journal.pone.0252267 (PMC8183983; doi:10.1371/journal.pone.0252267)
Supplement: S1 Table — 1 p-value-based on the chi-square, 2 p-value-based on the independent sample t-test. (DOCX) [file pone.0252267.s001.docx]

**S1 Table - Comparison of baseline characteristics in 2007 of the study population and the group lost to follow-up in 2017**

| **Baseline characteristics in 2007** | 10 year follow-up completed  (n=2517) | Lost to  follow up  (n=238) | P-value |
| --- | --- | --- | --- |
| Male sex, n, (%) | 1132 (45.0) | 114 (47.9) | 0.383^1^ |
| Mean age(+SD) years | 53.67 (6.7) | 53.97 (6.8) | 0.515^2^ |
| Current smokers, n(%) | 395(15.7) | 42 (17.6) | 0.084^1^ |
| Mean total cholesterol (+SD),mg/dL | 212.65 (41.9) | 210.10(44.6) | 0.394^2^ |
| Mean Fasting Plasma Glucose (+SD), mg/dL | 118.86 (43.7) | 122.03(56.6) | 0.330^2^ |
| Mean systolic blood pressure (+SD),mmHg | 136.25 (22.3) | 135.91 (20.4) | 0.822^2^ |

^1^ p-value based on the chi square

^2^ p-value based on the independent sample t-test
